# Reshaping membranes to build mitochondrial DNA

**DOI:** 10.1371/journal.pgen.1008140

**Published:** 2019-06-06

**Authors:** David Pla-Martin, Rudolf J. Wiesner

**Affiliations:** 1 Center for Physiology and Pathophysiology, Institute of Vegetative Physiology, University of Köln, Köln, Germany; 2 Cologne Excellence Cluster on Cellular Stress Responses in Aging-associated Diseases (CECAD), University of Köln, Köln, Germany; 3 Center for Molecular Medicine Cologne, University of Köln, Köln, Germany; Stanford University School of Medicine, UNITED STATES

In contrast to textbook pictures of bean-shaped blobs, mitochondria form a dynamic network of interconnected tubules in many cell types that may fall apart into smaller individual units, which are ready not only for intracellular transport but also to succumb to autophagy or will, at some, point fuse again into the network. Mitochondrial dynamics has been a central theme in mitochondrial biology in the last decade, but its role is still not fully clear [1]. The major hypothesis is that the small units are monitored for their performance and are more likely to be degraded by autophagy—or specifically mitophagy—if they do not properly function [2]. Dynamin-related guanosine triphosphatases (GTPases) perform and regulate these processes: dynamin-related protein 1 (DRP1) mediates division, whereas mitofusin 1 and 2 (MFN1 and MFN2) and optic atrophy 1 (OPA1), respectively, mediate fusion of the outer versus inner membrane. These proteins are essential for development, and mutations are known to cause human diseases, underlining the importance of mitochondrial dynamics [3–5]. Changes of mitochondrial morphology are known to correlate with mitochondrial (dys)function, and also other diseases involving mitochondrial processes are characterized by reshaping, but still the molecular players linking morphology and function are not known.

**Fig 1:**
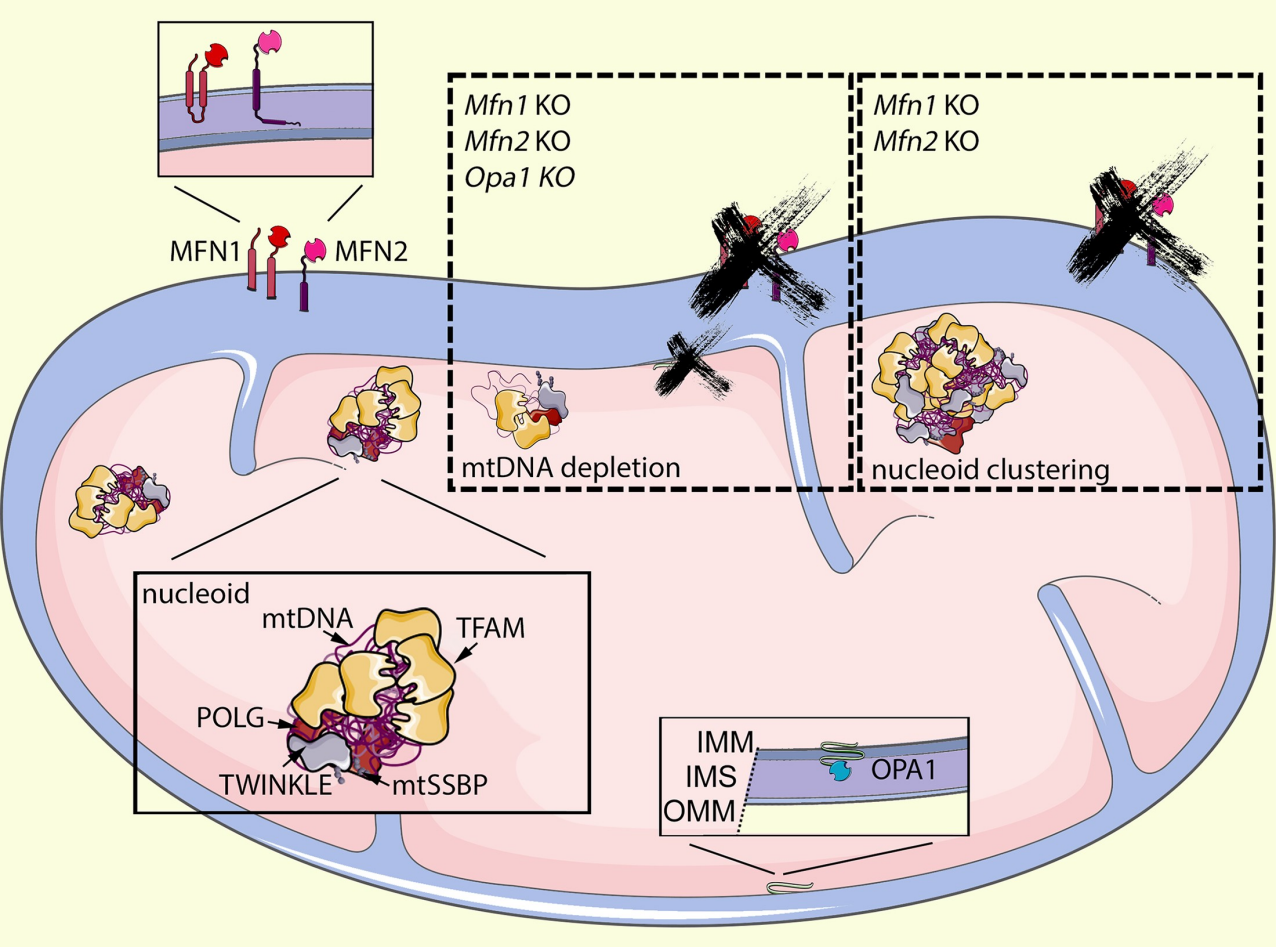
mtDNA copy number and distribution are regulated by the membrane fusion machineries. mtDNA is organized in nucleoids carrying approximately a thousand molecules of the packaging protein TFAM/mtDNA and the replisome machinery (POLG, TWINKLE, and mtSSBP1, among others). When OMM fusion machinery (MFN1 and MFN2) is mutated, losing its function, mtDNA clusters with reduced levels of POLG and mtSSBP1 and increased levels of TWINKLE, causing a replication rate decline and mtDNA depletion. The IMM fusion protein OPA1 is facing to the IMS and seems to not be involved in mtDNA clustering but is, as MFNs, required to maintain the replicative capacity of mtDNA [8]. IMM, inner mitochondrial membrane; IMS, intermembrane space; KO, knock-out; MFN, mitofusin; mtDNA, mitochondrial DNA; mtSSBP1, mitochondrial single-stranded binding protein; OMM, outer mitochondrial membrane; OPA1, optic atrophy 1; POLG, DNA polymerase gamma; TFAM, transcription factor A, mitochondrial; TWINKLE, mitochondrial helicase.

Even though most of the mitochondrial proteome is encoded by nuclear genes, mitochondria have their own DNA. Mitochondrial DNA (mtDNA) is a double-stranded, circular DNA that encodes 37 essential genes and is present in thousands of copies in cells and packaged into nucleoids together with proteins involved in protection (transcription factor A, mitochondrial [TFAM]; mitochondrial single-stranded binding protein [mtSSBP1]), transcription (RNA polymerase, mitochondrial [POLRMT]; TFAM; transcription factor B, mitochondrial [TFBM]) and replication (DNA polymerase gamma [POLG]; mitochondrial helicase [TWINKLE]), and many other roles (Fig 1) [6]. Mutations in genes related to mtDNA metabolism or within the mitochondrial genome have been extensively related not only to rare but fatal human diseases but also to the normal aging process [7]. Both mtDNA molecules containing mutations and/or reduced mtDNA copy numbers are, in many cases, consequences of replisome failure.

The fusion machinery is governed by two mitofusins for the outer membrane and OPA1 for the inner membrane. Physical interaction between mitofusins, forming homo- and heterodimers in opposing mitochondrial units, brings together the outer membranes, and by guanosine triphosphate (GTP) hydrolysis, membrane fusion takes place. Inner mitochondrial membrane (IMM) remodeling is controlled by OPA1, which contains a single-span transmembrane domain anchoring it in the inner membrane, leaving the protein facing the intermembrane space. Processing of OPA1 by proteolytic cleavage produces two proteins, known as small OPA1 (S-OPA1) and large OPA1 (L-OPA1), with several regulatory effects on mitochondrial function [1]. When mitochondria divide, mitochondrial–endoplasmic reticulum (ER) contacts are first established, which are proposed to serve as docking sites where mitochondria constrict [9]. The ER embraces mitochondria in a process orchestrated by Drp1 and perhaps also dynamin 2 (Dyn2), and constriction is facilitated by actin, together producing the force to divide the organelle.

It is thus evident that mitochondrial dynamics is directly linked to mitochondrial function and turnover. Despite ongoing changes in mitochondrial structure, cellular distribution, biogenesis, and degradation, mtDNA is maintained at stable levels. It was therefore surprising to observe a dysregulation in mtDNA homeostasis when interfering with mitochondrial dynamics, as they are, in principle, independent processes.

Whereas fission is not important to keep mtDNA levels constant [10], fusion seems to be critical for copy number maintenance [11–13]. Mitochondrial fission has been related to clearance of dysfunctional mitochondria [14]. Moreover, fission has been shown to be essential also for mtDNA distribution during mitochondrial division [9]. The reason why the ablation of fission does not affect mtDNA copy number remains unclear.

In the present work, Silva Ramos and colleagues [8] show for the first time a direct link between the dynamics machinery and mtDNA copy number. Using a battery of techniques in cardiomyocytes and immortalized MEFs (mouse embryonic fibroblasts), the authors link replication of mtDNA to the outer (MFN1 and MFN2) and inner (OPA1) membrane fusion proteins, but surprisingly, they show that nucleoid distribution only relies on the outer membrane fusion and fission proteins (Fig 1).

Traditionally, mtDNA depletion affecting the fusion machinery was related to increased mtDNA mutation rates [11]. However, when absolute levels were measured, very few molecules with deletions and point mutations were found, ruling out this as causative for depletion. On the other hand, mitochondrial dysfunction might cause an imbalance in important intermediate metabolites, especially the synthesis of pyrimidine nucleotides, which are necessary to make new mtDNA molecules, at the end causing mtDNA depletion. Here, the authors exclude both possibilities, despite some differences depending on the models they analyzed, but find an imbalance of components of the mtDNA replisome machinery when mitochondrial fusion is abolished.

Additionally, the authors further establish the outer mitochondrial membrane (OMM) as a key component for the distribution of mtDNA (Fig 1). Using superresolution microscopy, the authors show impressive clustering of nucleoids containing several molecules of mtDNA in the absence of outer membrane fusion, which could not have been resolved using conventional confocal microscopy. This aberrant aggregation, however, neither affects mitochondrial transcription activity nor is linked to the altered replisome composition.

The authors also show in a series of experiments the physiological relevance of these findings. The mixing of matrix components through IMM and OMM fusion is indispensable to keep mtDNA replicative rates high. Mitochondria, as a central hub in many metabolic pathways, need to be able to adapt, e.g., to changes in carbon source supply, and this is reflected in morphological changes [15, 16]. Beyond modulating mitochondrial network morphology during stress or in response to a changing metabolic environment, this work shows that mitochondrial fusion and fission also ensure proper content mixing required in order to maintain high mtDNA replicative capacity. The results the authors present suggest that mitochondrial content mixing induced by mitochondrial dynamics is necessary to maintain the delicate protein composition balance of the mitochondrial replisome. How mitochondrial outer and inner membrane fusion proteins affect replisome composition and why outer and not inner membrane fusion controls nucleoid distribution remains to be solved.
